# Multicenter Evaluation of the C6 Lyme ELISA Kit for the Diagnosis of Lyme Disease

**DOI:** 10.3390/microorganisms8030457

**Published:** 2020-03-24

**Authors:** Silvia Zannoli, Michela Fantini, Simona Semprini, Barbara Marchini, Barbara Ceccarelli, Monica Sparacino, Pasqua Schiavone, Anna Belgrano, Maurizio Ruscio, Martina Gobbetti, Maira Nicoletti, Eva Robatscher, Elisabetta Pagani, Vittorio Sambri

**Affiliations:** 1 Unit of Microbiology, The Great Romagna Area Hub Laboratory, 47522 Pievesestina di Cesena (FC), Italy; michela.fantini@auslromagna.it (M.F.); simona.semprini@auslromagna.it (S.S.); barbara.marchini@auslromagna.it (B.M.); barbara.ceccarelli@auslromagna.it (B.C.); monica.sparacino@auslromagna.it (M.S.); pasqua.schiavone@auslromagna.it (P.S.); vittorio.sambri@auslromagna.it (V.S.); 2 Azienda Sanitaria Universitaria Integrata - Trieste (ASUIT) Laboratory, 34149 Trieste, Italy; anna.belgrano@asuits.sanita.fvg.it (A.B.); maurizio.ruscio@asuits.sanita.fvg.it (M.R.); 3 Azienda Sanitaria Alto Adige-Bolzano Microbiology Laboratory, 39100 Bolzano, Italy; martina.gobbetti@sabes.it (M.G.); maira.nicoletti@sabes.it (M.N.); eva.robatscher@sabes.it (E.R.); elisabetta.pagani@sabes.it (E.P.); 4 Department of Experimental, Diagnostic and Specialty Medicine (DIMES), University of Bologna, 40138 Bologna, Italy

**Keywords:** lyme disease, C6, *Borrelia burgdorferi*

## Abstract

Lyme disease (LD), caused by infection with *Borrelia burgdorferi*, is the most common tick-borne infection in many regions of Eurasia. Antibody detection is the most frequently used laboratory test, favoring a two-step serodiagnostic algorithm; immunoenzymatic detection of antibodies to C6 has been shown to perform similarly to a standard two-step workflow. The aim of this study was the performance evaluation of the C6 Lyme ELISA kit compared to a standard two-step algorithm in three laboratories located in the northeastern region of Italy which cater to areas with different LD epidemiology. A total of 804 samples were tested, of which 695 gave concordant results between C6 testing and routine workflow (564 negative, 131 positive). Wherever available, clinical presentation and additional laboratory tests were analyzed to solve discrepancies. The C6 based method showed a good concordance with the standard two-step algorithm (Cohen’s κ = 0.619), however, the distribution of discrepancies seems to point towards a slightly lower specificity of C6 testing, which is supported by literature and could impact on patient management. The C6 ELISA, therefore, is not an ideal stand-alone test; however, if integrated into a two-step algorithm, it might play a part in achieving a sensitive, specific laboratory diagnosis of LD.

## 1. Introduction

Lyme disease (LD) is the most common tick-borne infection in many regions of Eurasia [1]. It is caused by the infection with *Borrelia burgdorferi* sensu lato spirochetes, which are transmitted by the bite of ixodid ticks where *Ixodes ricinus* is the main vector found in Europe [2].

Lyme borreliosis can be diagnosed through a combination of clinical observations and laboratory testing. The presence of erythema migrans (EM), an expanding circular skin rash, is considered sufficient to diagnose early Lyme disease in known endemic areas. However, later manifestations of LD are less specific, so highly reliable laboratory tests are necessary to support the diagnosis of suspected late LD [3]. Although the detection of *B. burgdorferi* DNA in samples such as skin biopsy or blood specimens would be an ideal choice in terms of specificity, the sensitivity of molecular testing is not sufficient for a negative result to rule out infection, especially in the cases of suspected neuroborreliosis [3,4].

Infection with other spirochetes or Epstein-Barr virus (EBV) and conditions of altered immunological reactivity, such as autoimmune diseases, may give false-positive results due to cross-reactions, an issue that was especially pronounced in first-generation LD diagnostic tests. The use of recombinant antigens or synthetic peptides has improved both the sensitivity and specificity of LD serological testing, but the issue is not yet solved [5].

To this purpose, laboratory testing in the USA [6] and in parts of Europe [7], favors a two-step serodiagnostic algorithm, where positive or indeterminate first-tier tests (typically a sensitive enzyme immunoassay or immunofluorescent assay) are retested by separate IgM and IgG immunoblots. The second-tier test must be positive for the sample to be considered seropositive [8].

The variable surface protein VlsE is an immunogenic molecule of *B. burgdorferi*, which has been proposed as a diagnostic antigen in enzyme-linked immunosorbent assay (ELISA) tests, both in its full-length form and the invariable region 6 (IR6) portion. Over the years, EIA detection of antibodies to C6, a synthetic peptide that reproduces the sequence of IR6, has been shown to perform similarly to standard 2-step workflow [9,10,11,12].

The aim of this study was the performance evaluation of the C6 Lyme ELISA kit (Immunetics Inc. Oxford Immunotec Ltd, Norwood, MA, USA) for the presumptive detection of IgG and IgM antibodies against *Borrelia burgdorferi*, compared to a standard two-step algorithm in three Italian laboratories which cater to areas with different LD epidemiology: the Romagna area, where LD reporting is scarce, but the presence of infected ticks has been ascertained [13]; the Giuliano-Isontina area (Friuli Venezia Giulia region), with a high LD prevalence and the highest infection risk in Europe [14]; the Alto Adige region, characterized by a marked difference in the seroprevalence (1.5%) in comparison to the nearby cross-border Tyrolean region (7.2%) [15]. A secondary aim was the evaluation of the assay as a potential diagnostic tool in a low endemicity region, such as Romagna.

## 2. Materials and Methods

The study was conducted on serum specimens deriving from the routine diagnostic procedures and collected from January to October 2019. The laboratories involved were the Great Romagna Area Hub Laboratory, Unit of Microbiology for the Romagna region, the Azienda Sanitaria Alto-Adige-Bolzano Microbiology Laboratory for the Alto Adige region, and the Azienda Sanitaria Universitaria Integrata di Trieste (ASUIT) Laboratory for the Giuliano-Isontina region.

The samples were anonymized using the current procedure (AVR-PPC P09, rev.2) checked by the local Romagna Ethical Board, keeping exclusively relevant data for the purpose of this study (age, sex, diagnosis, results of the two-tier routing algorithm). Each of the three laboratories involved in the study collected at least 200 samples routinely tested for anti-*B. burgdorferi* IgM and IgG antibodies, of which at least 50 were from patients presenting potential interfering factors that could potentially give false-positive results due to cross-reactions (*Treponema pallidum* or EBV infection, positivity to anti-nuclear antibodies indicating an autoimmune disease, ongoing pregnancy). Testing for interfering factors was conducted through using VCA IgM serology.

All three laboratories followed the same 2-step routing algorithm, used as a reference: a first-tier immunoassay with Liason XL CLIA (DiaSorin, Saluggia, Italy) and a confirmation of positivity using recomLine Borrelia IgG/IgM (Mikrogen Diagnostic GmbH, Neuried, Germany). Serum samples were then tested with the C6 Lyme ELISA kit (Immunetics Inc. Oxford Immunotec Ltd, Norwood, MA, USA).

While the specimens collected at the Great Romagna Area Hub Laboratory and the Azienda Sanitaria Alto Adige-Bolzano Microbiology Laboratory were selected consecutively amongst all samples with clinical suspicion of LD, the ASUIT Laboratory selected samples positive for anti-*Borrelia* antibodies to the 2-tier algorithm, whenever available. This was done to better evaluate discordant results between the standard workflow and C6 testing, as the Giuliano-Isontina region is characterized by the highest rate of LD prevalence in Italy.

Clinical presentation was requested along with laboratory testing in order to solve potential discrepancies between the two workflows.

To determine the concordance between the two workflows, Cohen’s κ coefficient was calculated in addition to the raw agreement rate, as the two-step diagnostic algorithm cannot be considered the gold standard. Given the differing LD epidemiology and collection criteria, κ was calculated both separately for each center and collectively. Data were processed using the STATA v14 software (College Station, TX, USA).

## 3. Results

A total of 804 samples were tested, of which 296 at the Great Romagna Area Hub Laboratory (Romagna area), 294 at the Azienda Sanitaria Alto-Adige-Bolzano Microbiology Laboratory (Alto Adige area), and 214 at the Azienda Sanitaria Universitaria Integrata di Trieste Laboratory (Giuliano-Isontina area). Table 1 illustrates the sample pool composition for each center.

Out of the total 804 samples, 695 (86.4%) gave concordant results between the two methods, of which 564 were negative and 131 positive. Table 2 illustrates the distribution of results at each participating center.

### 3.1. Analysis of Discordant Results

Wherever available, clinical presentation and additional exams were analyzed to solve discrepancies and discussed below.

#### 3.1.1. Romagna Area

The Great Romagna Area Hub Laboratory showed a total of 29 discrepancies. Of those, seven specimens testing negative for C6 were positive with the reference workflow, all presenting conditions which could potentially give rise to aspecific reactions (EBV infection, pregnancy, plasmacytoma). Twenty- two samples were positive with C6 testing and negative with the standard diagnostic algorithm; five of those were positive for interfering factors, while no additional clinical information was available for the remaining 17.

#### 3.1.2. Alto Adige Area

The Azienda Sanitaria Alto Adige-Bolzano Microbiology Laboratory tested 294 samples, 56 of which gave discordant results. Thirty-six specimens were positive for C6 but negative with the standard workflow; of these, seven of which were positive for interfering factors (EBV and *T. pallidum* infection); 11 of those samples could be demonstrated as actual positives pairing these results with clinical presentation, while clinical data was unavailable or not indicative of LD in the remaining cases.

In 20 cases, C6 EIA was negative while the 2-tier algorithm gave a positive result, of which five presented interfering factors; clinical data indicated eight of those as true positives.

#### 3.1.3. Giuliano-Isontina Area

The Azienda Sanitaria Universitaria Integrata di Trieste (ASUIT) Laboratory analyzed a total of 214 samples, selecting positives specimens for the standard diagnosis when possible. Unlike the two other laboratories, TS showed a higher number of C6 negative-routine diagnosis positive discordants (*n* = 15) compared to C6 positive-routine diagnosis negative (*n* = 9). All discrepancies were found in samples presenting no interfering factor. In each discordant group, three specimens could be identified as true positives when taking clinical presentation into account; in all other cases, data was unavailable or unhelpful to clarify the final serum status.

### 3.2. Concordance Evaluation

Raw agreement and Cohen’s κ were both calculated separately for each center and collectively (Table 3).

## 4. Discussion

The C6 Lyme ELISA kit showed a substantial agreement with the 2-tier workflow used in the routine. As expected, the concordance proved better in areas with a higher prevalence of positives, such as the Venezia Giulia region. It is interesting to note that despite showing the highest raw agreement, the PVS laboratory also showed the lowest κ. This is because κ is highest when the prevalence of positives is nearing 0.50 if raw agreement rates are equal. It follows that in low endemicity regions, such as Romagna, tests are less reliable, as a higher rate of accurate results is due to chance.

The highest number of discordant results was found in the C6 positive/2-tier negative group (*n* = 67). However, while clinical data could not solve all discrepancies, a greater proportion of true positives was found in the C6 negative/2-tier positive group. This would seem to point in the direction of a higher specificity of the currently recommended workflow, which is supported by the literature [16,17,18,19].

False-positive results are harmful to the patient in more than one way; on one hand, they may lead to superfluous antibiotic therapy, increasing the risk of antimicrobial resistance and adverse events; on the other hand, they might prevent a correct diagnosis and treatment, encouraging the attribution of non-specific symptoms to LD, to the extent of misdiagnosing malignancies as LD in extreme cases [20]. A higher cut-off value may be of help in increasing the specificity of the C6 testing, but that would affect sensitivity.

C6 ELISA, therefore, is not ideal as a stand-alone test; however, if integrated into a two-step algorithm, it might play a part in the laboratory diagnosis of Lyme disease.

On the other hand, false negatives resulting from the two-tier algorithm are common, resulting from factors such as patient seronegativity, and genetic diversity among disease-causing *Borrelia* strains. [21,22,23] ELISA assays are restricted in sensitivity as are confirmatory Western Blots [24] and about 20–30% of infected patients do not make detectable antibodies to *B. burgdorferi* [25]. To this purpose, laboratory diagnosis of LD must not overlook a judicious request of serological testing on the clinician’s part, in accordance with national guidelines. Nonetheless, the genetic diversity of *Borrelia* spp. has repercussions on test performance, as most commercially available LD kits are based on detection of just one strain, B31, making the CDC-endorsed two-step workflow highly specific for *B. burgdorferi* sensu stricto (Bbss), [26,27] sacrificing sensitivity and focusing on a narrow case definition of LD. Testing for *Borrelia* spirochetes should encompass the spectrum of *Borrelia* organisms capable of causing disease, including Bbss, *B. burgdorferi* sensu lato (Bbsl) and the Relapsing Fever *Borrelia* (RFB). Ideally, testing should detect both LD and RFB and should differentiate between the two.

Recently, modified two-tiered testing (MTTT) algorithms that utilize two sequential first-tier tests and eliminate immunoblotting have been evaluated. Specifically, all MTTTs resulted in a significantly higher proportion of correct classifications than standard two-tier testing [28,29].

It must always be kept in mind that a laboratory diagnosis of LD is imperfect and no test available at the moment is reliably sensitive and specific.

## Figures and Tables

**Table 1:** pool composition.

	Clinical Suspicion of LD – No Interfering Factors	*T. pallidum* Infection	EBV Infection	Anti-Nucleus Antibodies (ANA) Positivity	Ongoing Pregnancy	Total
Romagna	236	15	15	15	15	296
Alto Adige	240	10	14	10	20	294
Giuliano-Isontina	164	7	14	16	13	214
Total	640	32	43	41	48	804

**Table 2:** Distribution of results per center.

	Concordant Negative	Concordant Positive	C6 Pos/Routine Neg	C6 Neg/Routine Pos	Total
Romagna	254	13	22	7	296
Alto Adige	176	62	36	20	294
Giuliano-Isontina	134	56	9	15	214
Total	564	131	67	42	804

**Table 3:** Raw agreement and Cohen’s κ per center.

	Raw Agreement	Cohen’s κ	Strength of Agreement
Romagna	90.2%	0.423	Moderate
Alto Adige	81.0%	0.553	Moderate
Giuliano-Isontina	88.8%	0.742	Substantial
Overall	86.4%	0.619	Substantial
